# p53-dependent c-Fos expression is a marker but not executor for motor neuron death in spinal muscular atrophy mouse models

**DOI:** 10.3389/fncel.2022.1038276

**Published:** 2022-11-07

**Authors:** Jannik M. Buettner, Leonie Sowoidnich, Florian Gerstner, Beatriz Blanco-Redondo, Stefan Hallermann, Christian M. Simon

**Affiliations:** ^1^Carl-Ludwig-Institute for Physiology, Leipzig University, Leipzig, Germany; ^2^Division of General Biochemistry, Rudolf Schönheimer Institute of Biochemistry, Medical Faculty, Leipzig University, Leipzig, Germany

**Keywords:** spinal muscular atrophy, p53, motor circuit pathology, motor neuron death, c-fos, SMA mouse models

## Abstract

The activation of the p53 pathway has been associated with neuronal degeneration in different neurological disorders, including spinal muscular atrophy (SMA) where aberrant expression of p53 drives selective death of motor neurons destined to degenerate. Since direct p53 inhibition is an unsound therapeutic approach due carcinogenic effects, we investigated the expression of the cell death-associated p53 downstream targets *c-fos*, *perp* and *fas* in vulnerable motor neurons of SMA mice. Fluorescence *in situ* hybridization (FISH) of SMA motor neurons revealed *c-fos* RNA as a promising candidate. Accordingly, we identified p53-dependent nuclear upregulation of c-Fos protein in degenerating motor neurons from the severe *SMN*Δ*7* and intermediate *Smn^2*B/*–^* SMA mouse models. Although motor neuron-specific *c-fos* genetic deletion in SMA mice did not improve motor neuron survival or motor behavior, p53-dependent c-Fos upregulation marks vulnerable motor neurons in different mouse models. Thus, nuclear c-Fos accumulation may serve as a readout for therapeutic approaches targeting neuronal death in SMA and possibly other p53-dependent neurodegenerative diseases.

## Introduction

Neurodegenerative disorders such as Parkinson’s disease (PD), Alzheimer’s disease (AD), Huntington’s disease (HD), and motor neuron diseases result from death of a subset of dopaminergic, hippocampal, striatal, and motor neurons, respectively (Donev et al., 2009; Bano et al., 2011; Michel et al., 2016; Wirth, 2021). However, the mechanisms by which mutations of ubiquitously expressed genes induce selective death of a distinct group of neurons remain unclear. Therefore, an important step toward developing therapeutic strategies for neurodegenerative disorders is to reveal possibly shared molecular pathways responsible for neuronal death. There is mounting evidence that the activation of the tumor suppressor p53 pathway contributes to neuronal death in PD, AD, HD, and motor neuron diseases such as amyotrophic lateral sclerosis (ALS) and spinal muscular atrophy (SMA) (Zhang et al., 2002; Chang et al., 2012; Qi et al., 2016; Simon et al., 2017; Vogt et al., 2018; Buettner et al., 2021; Maor-Nof et al., 2021).

Spinal muscular atrophy is an autosomal recessive neurological disorder in children (Feldkotter et al., 2002) that is caused by homozygous loss of the *survival motor neuron 1* (*SMN1*) gene with the retention of the hypomorphic *SMN2* gene leading to reduced levels of the ubiquitously expressed SMN protein (Tisdale and Pellizzoni, 2015; Wirth, 2021). The *SMN2* copy number and the resulting functional amount of SMN protein define the onset and severity of SMA, which can be replicated in the severe *SMN*Δ*7* and intermediate *Smn^2*B/*–^* SMA mouse models (Le et al., 2005; Bowerman et al., 2012; Tisdale and Pellizzoni, 2015). SMA patients and mouse models develop a progressive weakness of proximal muscles caused by selective loss of the corresponding innervating motor neurons (Burghes and Beattie, 2009; Mentis et al., 2011; Buettner et al., 2021). Several studies using cell cultures and SMA mouse models have demonstrated that motor neurons die in a cell-autonomous matter (Martinez et al., 2012; Simon et al., 2016; Fletcher et al., 2017). Importantly, transcriptional profiling of vulnerable motor neurons identified a robust activation of the p53 pathway, and its genetic and pharmacological inhibition prevented motor neuron death in severe *SMN*Δ*7* and intermediate *Smn^2*B/*–^* SMA mouse models (Murray et al., 2015; Simon et al., 2017, 2019; Van Alstyne et al., 2018; Nichterwitz et al., 2020; Buettner et al., 2021), suggesting p53 as a main driver of motor neuron death in SMA.

Due to the role of p53 in tumor suppression (Dai and Gu, 2010), its direct long-term inhibition is unlikely to be a viable therapeutic target. In contrast, the identification of p53-downstream effectors could yield novel targets for not only SMA, but also for other p53-associated neurological diseases such as PD, AD, HD, and ALS. Previous studies reported the activation of the Fas death receptor, a known p53-dependent mediator of the apoptotic pathway, in motor neurons from SMA patients and the SMA mouse models (Sareen et al., 2012; Wang et al., 2014; Murray et al., 2015; Van Alstyne et al., 2018). Transcriptional profiling of SMA motor neurons identified several upregulated p53 effectors (Simon et al., 2017), of which most are associated with suppression of apoptosis, tumor growth, oxidative stress response or cell cycle arrest, but two others, *perp* and *c-fos*, have been linked to cell death (Attardi et al., 2000; Seoane et al., 2002; Ihrie et al., 2003; Hartland et al., 2009; Sareen et al., 2012; Miyake et al., 2015; Murray et al., 2015; Simon et al., 2017; Han et al., 2018). Perp triggers apoptosis in non-neuronal tissues and mediates γ-irradiation-induced neural precursors death of the subventricular zone (Attardi et al., 2000; Ihrie et al., 2003), while upregulated c-Fos induces neuronal cell death in a p53-dependent manner (Smeyne et al., 1993; Preston et al., 1996; Elkeles et al., 1999; Xu et al., 2005; Chen et al., 2015).

In this study, we sought to investigate whether *fas*, *perp* or *c-fos* mediate p53-dependent motor neuron death in SMA mice. Time course analysis identified a correlation of motor neuron death with *c-fos* RNA and protein upregulation. While p53-dependent c-Fos protein expression does not execute degeneration, it marks vulnerable motor neurons in severe *SMN*Δ*7* and intermediate *Smn^2*B/*–^* mouse models. Our results reveal p53-dependent c-Fos expression as a novel marker for neuronal death during therapeutic approaches in SMA and possibly other p53-dependent neurodegenerative diseases.

## Materials and methods

### Animal procedure and motor phenotype

Breeding and experiments were performed in the animal facilities of the Faculty of Medicine, University of Leipzig according to European (Council Directive 86/609/EEC) and German (Tierschutzgesetz) guidelines for the welfare of experimental animals and the regional directorate (Landesdirektion) of Leipzig. Mice were housed in a 12 h/12 h light/dark cycle with access to food and water *ad libitum*. The original breeding pairs for *SMN*Δ*7* (*Smn*^±^; *SMN2*^+/+^; *SMN*Δ*7*^+/+^) mice (Jax stock #005025) on *FVB* background were obtained from Jackson Laboratory. The *Smn^2*B/*–^* mice on a *C57BL6* background were generated by the Kothary lab (Bowerman et al., 2012) and provided by Dr. Kothary and Dr. Raoul following the breeding scheme as described previously (Bowerman et al., 2012). *SMN*Δ*7* line was crossed with *ChAT-Cre* (Jax stock #006410) and *c-Fos*^fl/fl^** (Fleischmann et al., 2003) mouse lines (both on a *C57BL/6* background) provided by Dr. Erwin Wagner and Dr. Latifa Bakiri to generate *Smn*^–/^*^–^*; *SMN2*^+/+^; *SMN*Δ*7*^+/+^; *ChAT-Cre*^±^; *c-Fos*^fl/fl^** (SMA *c-fos^Δ^ *^MN^**). The resulting SMA *c-fos^Δ^ *^MN^** line was maintained on a mixed *C57BL/6*;*FVB* background. Control animals were littermates of mutants from each individual SMA mouse line. As reported previously, the controls were for *Smn^2*B/*–^* line = *Smn*^2*B*/+^ (Bowerman et al., 2012), *SMN*Δ*7* = *Smn*^+/+^; *SMN2*^+/+^; *SMN*Δ*7*^+/+^ (Le et al., 2005) and *Smn*^–/^*^–^*; *SMN2*^+/+^; *SMN*Δ*7*^+/+^; *c-Fos*^fl/fl^** with either *Chat-Cre*^+/–^ = SMA *c-fos^Δ^ *^MN^** or *ChAT-Cre^–/–^* = SMA *c-fos*^fl/fl^** were used, respectively.

Primers for genotyping: *SMN*Δ*7*: forward sequence (5′–3′) = GATGATTCTGACATTTGGGATG, reverse sequences (5′–3′) = TGGCTTATCTGGAGTTTCACAA and GAGTAA CAACCCGTCGGATTC (wild-type band: 325 bp, mSmn ko: 411 bp). *Smn^2*B/*–^*: forward sequence (5′–3′) = TTTGG CAGACTTTAGCAGGGC, reverse sequence (5′–3′) = AA CTCCGGGTCCTCCTTCCT (wild-type band: 500 bp, mutant: 700 bp). *c-Fos*: forward sequence (5′–′) = GAGTCGCTAA CTAGAGTTTGGGAGG, reverse sequence (5′–3′) TGTCTTCC TGTATGCACCTCATCG (wild-type band: 335 bp, flox c-Fos: 400 bp, deleted c-Fos: 500 bp). Chat-Cre: common reverse sequence (5′–3′) = GGCCACTTAGATAGATAATGAGG GGCTC; wild-type forward primer (5′–3′) = GTTTGCAG AAGCGGTGGG, *ChAT-Cre* transgene forward sequence (5′–3′) = TCGCCTTCTTGACGAGTTCTTCTG (wild-type = 272 bp, transgene = 350 bp).

Pifithrin-α (PFT) was dissolved in DMSO and delivered daily at a concentration of 2.2 mg/kg by IP injection starting at P0 as described previously (Simon et al., 2017). For AAV9 gene delivery, P0 mice were anesthetized by isoflurane inhalation and injected in the right lateral ventricle of the brain with ∼1 × 10^11^ genome copies of AAV9 vectors (Cre and GFP, VectorBuilder) in a phosphate buffered saline (PBS) solution containing a vital dye (Fast Green; Sigma-Aldrich) as described previously (Simon et al., 2017).

Mice from all experimental groups were monitored daily, body weight measurements and the righting reflex were timed and averaged as described previously (Mentis et al., 2011). Mice with a 25% reduction of body weight and an inability to right were euthanized to comply with German guidelines for the welfare of experimental animals. Righting time was defined as the time for the pup to turn over on all its four limbs after being placed on its back. The cut-off time for the righting reflex was 60 s to comply with German guidelines for the welfare of experimental animals. Approximately equal proportions of mice of both sexes were used and aggregated data are presented since gender-specific differences were not found nor have they been previously reported.

### Immunohistochemistry

For immunostainings, the spinal cords were either natively dissected with 10°C oxygenated artificial cerebrospinal fluid (aCSF) or perfused with 1× PBS and 4% paraformaldehyde (PFA) following 4% PFA postfixation overnight at 4°C. On the following day, the spinal cords were taken out and the specific segments of interest were identified by the ventral roots. For detailed methodology, please see Buettner et al. (2022). Briefly, subsequently single segments were embedded in warm 5% agar and serial transverse sections (75 μm) were cut at the vibratome. The sections were blocked with 5% normal donkey serum in 0.01 M PBS with 0.3% Triton X-100 (PBS-T; pH 7.4) for 90 min and incubated overnight at room temperature in different combinations of the primary antibodies. Vesicular glutamate transporter 1 (VGluT1) antibodies were used as a marker for proprioceptive synapses, ChAT antibodies as motor neuron markers (Table 1: List of antibodies). The following day, after six times of 10 min PBS washes, secondary antibody incubations were performed for 3 h with the appropriate species-specific antiserum coupled to Alexa488, Cy3 or Alexa647 (Jackson labs) diluted at 1:1,000 in PBS-T. After secondary antibody incubations, the sections were washed six times for 10 min in PBS and mounted on slides and cover-slipped with a Glycerol:PBS (3:7) solution.

**TABLE 1:** List of antibodies.

Name	Company	Cat #	Host	Application	Dilution
c-Fos	Abcam	AB190289	Rabbit	IF	1:20,000
p53	Leica Novocastra	NCL-p53-CM5p	Rabbit	IF	1:1,000
p53	Cell Signaling	2524S	Mouse	IF	1:2,000
ChAT	Millipore	AB144P	Goat	IF	1:500
VGluT1	Synaptic Systems	135 304	Guinea pig	IF	1:5,000
SV2	DSHB	SV2, concentrate	Mouse	IF	1:500
Neurofilament	DSHB	2H3, concentrate	Mouse	IF	1:500
Bungarotoxin	Invitrogen	B35451	N/A	IF	1:500
c-Fos	Abcam	AB190289	Rabbit	WB	1:2,000
Tubulin	Sigma	T9026	Mouse	WB	1:5,000

For immunostaining of neuromuscular junctions (NMJs), mice were sacrificed or perfused and the muscle was dissected and immediately fixed with 4% PFA overnight. After fixation, single muscle fibers were teased and washed three times in PBS for 10 min each followed by staining of the postsynaptic part of the NMJ with α-bungarotoxin (BTX) Alexa Fluor 555 in PBS for 20 min. Subsequently, the muscle fibers were washed five times in PBS for 10 min and blocked with 5% donkey serum in 0.01 M PBS with 0.3% Triton X-100 (PBST) for 1 h. Mouse anti-neurofilament and anti-SV2 antibodies to immunolabel the presynaptic aspect of the NMJ were applied in blocking solution overnight at 4°C (Table 1: List of antibodies). The muscle fibers were then washed three times for 10 min in PBS. Secondary antibodies were applied for 1 h in PBS-T at room temperature. Finally, the muscle fibers were washed three times in PBS for 10 min and mounted on slides covered with Glycerol:PBS (3:7) as established in Simon et al. (2010).

### Confocal microscopy and analysis

Spinal cord sections were imaged using SP8 Leica confocal microscopes. Sections were scanned using a 20×, 40×, or 63× objective. Motor neurons were scanned with a 20× objective and were counted off-line from *z*-stack images (collected at 4-μm-intervals in the *z*-axis) from an entire spinal cord segment. Control and mutant spinal cord segments were measured from one ventral root to the other with a 10× light microscope to ensure consistency. All control and mutant segments at the same age were of equal length, except for the P26 *Smn^2*B/*–^* mutant L1 segment, which was shorter than control age-matched littermates (Buettner et al., 2021). Only ChAT+ motor neurons located within the ventral horn containing the nucleus were counted to avoid double counting from adjoining sections.

Quantitative analysis of VGluT1+ proprioceptive synapses on motor neurons was performed on image stacks of optical sections scanned using a 40× oil (numerical aperture: 1.3) or 63× glycerol (numerical aperture: 1.4) objective throughout the whole section thickness at 0.4 μm *z*-steps to include the whole cell body and dendrites of ChAT+ motor neurons as detailed described in Buettner et al. (2022). The number of VGluT1+ were counted over the entire surface of the motor neuron soma as well as on primary dendrites for VGluT1+ synapses up to a distance of 50 μm from the soma using Leica LASX software as previously described (Mentis et al., 2011; Buettner et al., 2021). VGluT1 density from at least 10 motor neurons per mouse were quantified.

For the analysis of muscle innervation, a minimum of 200 randomly selected NMJs per muscle sample were scanned at 3 μm *z*-steps and quantified for each biological replicate. Only BTX+ endplates that lack pre-synaptic coverage by both SV2 and NF were scored as fully denervated as previously described (Simon et al., 2010). JMB and LS conducted the morphometric measurements and quantifications (motor neuron counts, number of c-Fos/p53+ motor neurons, synaptic numbers) in a blinded manner. Genotypes of age-matched littermates were revealed after quantification was completed.

### Fluorescence *in situ* hybridization

Mice were sacrificed and perfused with 4% PFA and the spinal cords were postfixed at 4°C overnight. On the next day, dissected L1 spinal cord segments (Buettner et al., 2022) were cryoprotected in 15% sucrose containing 1× PBS solution at first for 4 h and afterward in 30% Sucrose 1× PBS solution at 4°C overnight. The tissue was then embedded in Sakura Tissue-Tek O.C.T.™ Compound and frozen within 2-methylbutan cooled by liquid nitrogen. L1 segments were cut at a Leica CM3050S cryostat at 20 μm thickness and mounted on ThermoScientific Superfrost Plus slides. For FISH, the RNAscope Multiplex Fluorescent Reagent Kit v2 (Cat. No. 323100) from Advanced Cell Diagnostics (ACD) was used and the corresponding protocol was followed. Three housekeeping genes (PolR2A as low, PPIB as medium and UBC as high expresser) were tested for establishing the protocol and checking mRNA integrity on 3T3 cells (provided in Kit) and murine spinal cords. For *perp, c-Fos* and *fas* mRNA detection the following RNAscope^®^ probes were applied: Mm-Perp catalog #515481, Mm-Fos-C2 catalog #316921-C2, and Mm-Fas catalog #43956. For subsequent ChAT immunostaining, the slides were blocked at room temperature for 1 h with 5% normal donkey serum in PBS-T before incubation with a ChAT antibody diluted 1:500 in blocking solution overnight at 4°C. The following day, the slides were washed three times with 1× PBS for 10 min each and then incubated for 1 h at room temperature with an anti-goat-AlexaFluor647 secondary antibody. Finally, the slides were washed three times with 1× PBS for 10 min each and then cover-slipped using ProLong Gold Antifade Mountant.

The slides were imaged using a Leica TCS SP8 inverted confocal microscope. Slides were scanned using a 20× immersion oil objective. The images were taken with a *z*-step size of 3 μm. For analysis the Fiji Image J and Quantitative Pathology (QuPath) software was used. First, a maximum projection of all *z*-panels was created for each channel in the Fiji Image J software. To reduce the signal to noise ratio in the RNA probe channels, a background subtraction was performed on the maximum-projection of each probe channel (*fas* or *perp* and *c-fos*). Therefore, the standard deviation of each maximum projection was measured using the Fiji Image J software. The individual standard deviation for each maximum projection was multiplied with the factor three and subtracted from the image to eliminate most of the background signal. Then the modified maximum projections were combined again and exported to the QuPath software. The area of the motor neuron soma was measured by the software via the ChAT signal. Afterward the area of the signal for the stained probes within each measured motor neuron was estimated. The data was then transferred to an Excel sheet and the percentage of the probe signal covering the ChAT area of each motor neuron was calculated. For each probe and time point the mean of coverage was calculated for all control animals and the mean of the standard deviation was added to generate a threshold. This threshold was applied to all animals of each time point and probe. Every motor neuron with a signal of the individual probe above the threshold was scored as an expressing motor neuron. 11–30 motor neurons per animal were analyzed and the mean per animal was calculated. Finally, all percentages for each analyzed motor neuron of the corresponding animals were pooled together for each probe and time point and compared using the GraphPad Prism 9 software.

### Western blot analysis

Whole spinal cords were prepared in RIPA buffer (Thermofisher, catalog number: 89900) and resolved using the NuPAGE^®^ precast gel system (Thermofisher, catalog number: 89900) by SDS-PAGE. Extracts (20 μg) were run on Novex^®^ Bis-Tris 12% gels and transferred onto an iBlot2 transfer stack nitrocellulose membrane (Thermofisher, catalog number: 89900) using the iBlot2 Dry Blotting system unit (Thermofisher, catalog number: 89900). After protein transfer, the membranes were blocked for 1 h in 5% non-fat dry milk prepared in 1× PBS with 0.1% Tween-20. The membranes were then incubated with the corresponding antibodies overnight (see Table 1: List of antibodies). Thereafter, the membranes were incubated with IRDye 680RD or 800CW secondary antibodies (Li-cor) followed by visualization using a near-infrared imager (Odyssey; Li-cor) (Blanco-Redondo et al., 2019). Band intensities were analyzed by Fiji ImageJ.

### Electrophysiology

To record NMJ function, we conducted the experiment as previously described (Fletcher et al., 2017; Simon et al., 2017). The animals were decapitated, the organ-free carcass which exposes the spinal column was ventral side-up pinned down and the spinal cord without the ventral roots was dissected out under cold (∼10°C) aCSF containing 128.35 mM NaCl, 4 mM KCl, 0.58 mM NaH_2_PO_4_.H_2_0, 21 mM NaHCO_3_, 30 mM D-Glucose, 1.5 mM CaCl_2_.H20, and 1 mM MgSO_4_.7H_2_0. The preparation of the remaining carcass with the intact ventral roots in connectivity with the quadratus lumborum (QL) muscle was then transferred to a customized recording chamber and perfused continuously with oxygenated (95%O_2_/5%CO_2_) aCSF (∼13 ml/min). Motor neuron axons in the ventral root L1 supplying the QL muscle were stimulated by drawing the ventral root into a suction electrode and recorded the compound muscle action potential (CMAP) from the muscle using a concentric bipolar electrode. The extracellular potentials were recorded (DC recordings—3 kHz, Cyberamp, Molecular Devices) in response to a brief (0.2 ms) stimulation (A365, current stimulus isolator, WPI, Sarasota, FL) of the L1 ventral root. Recordings were fed to an A/D interface HEKA EPC10/2 amplifier (HEKA Elektronik, Lambrecht/Pfalz, Germany) and acquired with HEKA Patchmaster (HEKA Electronics) amplifier at a sampling rate of 20 kHz. Data were analyzed off-line using HEKA Patchmaster (HEKA Electronics). The temperature of the aCSF solution ranged between 21 and 23°C. L1 motor neuron axons were stimulated with five stimuli at 1 Hz for peak-to-peak measurements of the maximum CMAP amplitude from averages of five recordings. The latency was measured from the visually identified beginning of the stimulus artifact and the visually identified onset of the response. Conduction velocity was calculated as previously described (Kong et al., 2021).

### Statistics

Results are expressed as mean + standard error of the mean (SEM) from at least three or more animals per experimental group. Shapiro–Wilk-Test has been used to test for normal distribution. Each applied statistical test is listed in the appropriate figure legend. *n* values indicate number of animals. GraphPad Prism 9 was used for all statistical analyses and *p*-values are indicated as follows: *p < 0.05; **p < 0.01; ***p < 0.001 ****p < 0.0001.

## Results

### Upregulated nuclear c-Fos expression marks motor neuron death in the severe *SMN*Δ*7* mouse model

**FIGURE 1:**
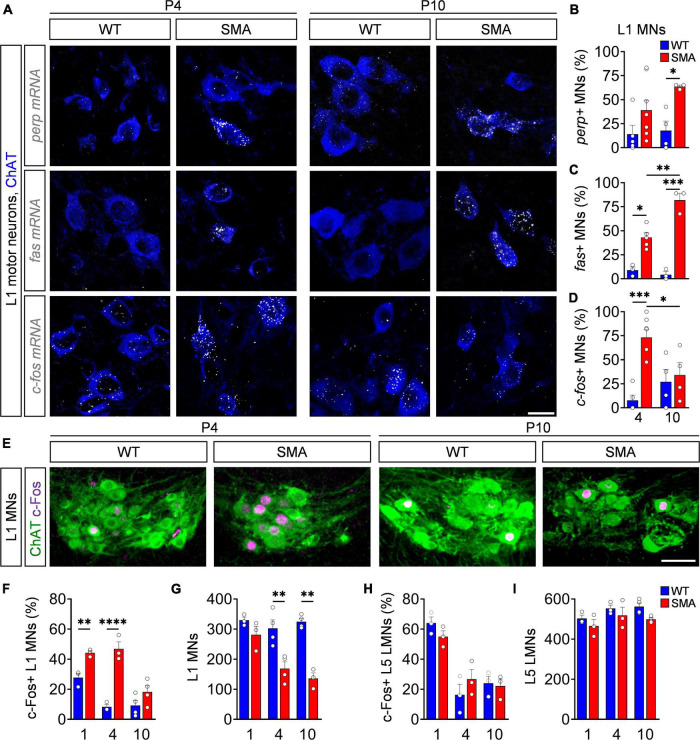
c-Fos upregulation reliably marks motor neuron degeneration in a severe spinal muscular atrophy (SMA) mouse model. **(A)** Fluorescence *in situ* hybridization (FISH) analysis of *perp*, *fas* or *c-fos* mRNA (white) expression with ChAT (blue) immunostaining as a motor neuron (MN) marker of L1 spinal cord segment from P4 and P10 control wild-type (WT) and *SMN*Δ*7* mutant (SMA) mice. Scale bar = 20 μm. **(B–D)** Quantification of *perp*, *fas*, and c-*fos* mRNA positive MNs from the same groups as in panel **(A)** (for details see section “Fluorescence *in situ* hybridization”). *n* = 3–8 per genotype. For each animal 11–30 motor neurons were analyzed. **(E)** Immunostaining of c-Fos (magenta) and L1 ChAT + motor neurons (green) of spinal cords from the same groups as in panel **(A)**. Scale bar = 50 μm. Quantification of c-Fos positive L1 MNs in percent **(F)**, number of L1 MNs **(G)**, c-Fos + L5 lateral motor neurons (LMNs) in percent **(H)** and number of L5 LMNs **(I)**. *n* = 3–4 per genotype. Statistics: two-way ANOVA with Tukey’s correction for panels **(B,C,F–I)**. Each data point (n) represents one animal. Data are presented as mean ± SEM. Asterisks on top of bars without horizontal line indicate the significance compared to another group. *p < 0.05; **p < 0.01; ***p < 0.001, ****p < 0.0001.

Based on the known functions of p53-dependent genes upregulated in SMA mice, *c-fos*, *fas*, and *perp* are the priority candidates for being potential executors of motor neuron cell death (Murray et al., 2015; Simon et al., 2017). Previous studies demonstrated selective death of lumbar L1 motor neurons that innervate proximal muscles in the severe *SMN*Δ*7* SMA mouse model, which starts shortly after birth peaking at postnatal (P) day 4, while almost no further motor neuron death occurs until end-stage (P10) (Mentis et al., 2011; Simon et al., 2017; Buettner et al., 2021). To correlate the expression of *c-fos*, *fas*, and *perp* with motor neuron death, we performed FISH for each mRNA candidate on spinal cord tissue. To ensure proper execution of the FISH protocol and mRNA integrity, we successfully tested the expression of provided control mRNAs in 3T3 cell lines and spinal cord tissue (Supplementary Figure 1A). Next, we co-stained against *c-fos*, *perp* or *fas* mRNA in combination with antibodies against choline acetyltransferase (ChAT) to visualize L1 motor neurons in spinal cord sections of *SMN*Δ*7* (SMA) mice and control wild-type (WT) littermates at a time point with high (P4) and no/little motor neuron death (P10). Expression of *perp* and *fas* mRNA is increased in SMA mice at P4 but elevates further at P10 (Figures 1A–C), when the process of L1 motor neuron death is already completed (Mentis et al., 2011; Simon et al., 2017; Buettner et al., 2021). In contrast, *c-fos* expression is strongly upregulated in SMA at P4, but decreases to WT level at P10 (Figures 1A,D). Thus, only *c-fos* mRNA expression timely correlates with motor neuron death in a severe SMA mouse model.

To investigate the c-Fos protein expression, we performed immunohistochemistry and western blot analysis of spinal cord tissue from WT and SMA mice at different time points. Applied antibodies against c-Fos and ChAT revealed no difference between nuclear c-Fos expression in non-motor neuron cells throughout the spinal cord at P4 and P10 (Supplementary Figures 1B,C). Accordingly, the total amount of c-Fos in P4 lumbar spinal cords of SMA mice was not elevated in western blot analysis (Supplementary Figure 1D). However, while c-Fos is expressed at basal levels in WT mice, its expression significantly increased at P1 and peaks at P4 in vulnerable L1 SMA motor neurons, while no difference was found between mutants and controls at P10 (Figures 1E,F), correlating nuclear c-Fos expression pattern with the time course of L1 motor neuron death (Figures 1E,G). In agreement, c-Fos was not upregulated in the L5 lateral motor neurons (LMN) of SMA mice at any investigated time point which correlates with the lack of death in this resistant motor neuron pool (Figures 1H,I and Supplementary Figure 1E). These results reveal a strong correlation of the amount of c-Fos upregulation and occurring motor neuron death in vulnerable SMA motor neuron pools.

### Nuclear c-Fos upregulation in spinal muscular atrophy motor neurons is p53-dependent

**FIGURE 2:**
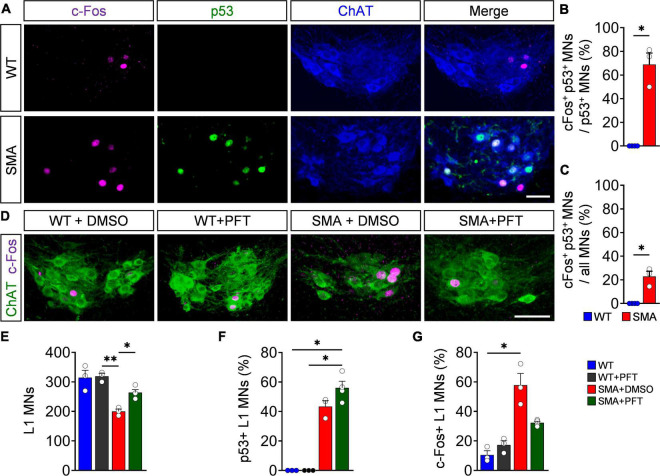
p53 drives c-Fos upregulation in spinal muscular atrophy (SMA) motor neurons. **(A)** Immunostaining of c-Fos (magenta), p53 (green), and ChAT + (blue) motor neurons (MNs) of L1 spinal segments from P4 control wild-type (WT) and *SMN*Δ*7* mutant (SMA) mice. Scale bar = 50 μm. Quantification of MNs coexpressing both c-Fos and p53 in comparison to p53 positive MNs **(B)** or **(C)** all MNs in% from the same groups as in panel **(A)**. *n* = 3 per genotype. **(D)** Immunostaining of c-Fos (magenta) and L1 ChAT + motor neurons (green) of spinal cords from P4 WT and SMA animals treated chronically with DMSO or Pifithrin-α (PFT). Scale bar = 50 μm. Number of L1 MNs **(E)**, percentage of p53 **(F)**, and c-Fos positive **(G)** MNs. *n* = 3–4 per genotype. Statistics: Mann–Whitney test for panels **(B,C)**, Kruskal–Wallis test for panel **(F)** and one-way ANOVA with Tukey’s correction for panels **(E,G)**. Each data point (n) represents one animal. Asterisks on top of bars without horizontal line indicate the significance compared to another group. *p < 0.05; **p < 0.01.

To validate whether c-Fos expression is induced by activation of the p53 pathway in SMA, we stained L1 spinal segments from P4 SMA and WT mice for p53 and c-Fos in ChAT+ motor neurons. 20% of all SMA motor neurons expressed c-Fos without being p53+ which equals the amount of c-Fos + WT motor neurons (Supplementary Figure 2C), suggesting a common baseline expression of c-Fos in both genotypes. Intriguingly, additionally 20% of all P4 SMA motor neurons co-express c-Fos and p53 and over 70% of p53+ motor neurons exhibit c-Fos colocalization (Figures 2A–C), while p53 and c-Fos coexpression in non-motor neuron cells throughout the spinal cord of P4 and P10 SMA mice was almost completely absent (Supplementary Figures 2A,B), suggesting a specific p53-dependent c-Fos upregulation in SMA motor neurons. To test direct c-Fos-dependency of p53 activation, SMA mice were daily treated with PFT, a chemical p53 inhibitor which reliably inhibits the transcriptional activity of p53 and its downstream effectors in SMA mouse models (Simon et al., 2017, 2021; Buettner et al., 2021). In agreement, PFT treatment prevented motor neuron death in P4 SMA mice without altering p53 expression itself (Figures 2D–F). Importantly, p53 inhibition resulted in significant decrease of c-Fos in PFT-treated P4 SMA mice compared to DMSO-treated mutant littermates, while PFT has no effect on c-Fos expression in WT mice (Figures 2D,G). These experiments demonstrate that c-Fos upregulation in SMA is indeed p53-dependent.

### Deletion of c-Fos in motor neurons does not prevent their death in spinal muscular atrophy mice

**FIGURE 3:**
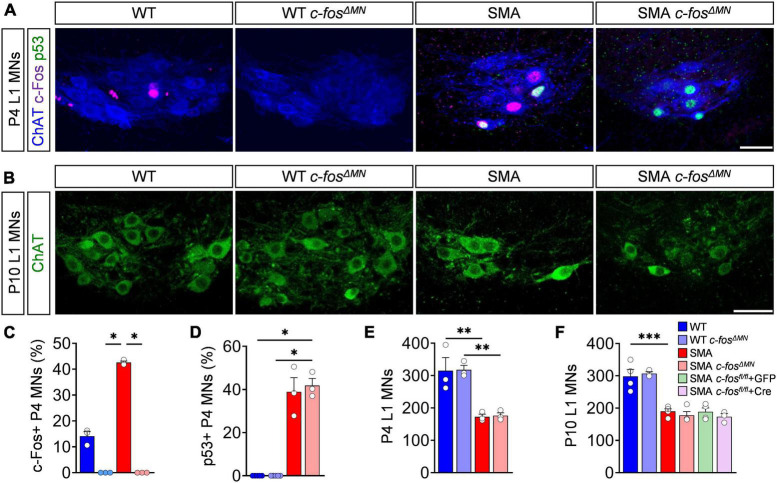
c-Fos deletion in spinal muscular atrophy (SMA) motor neurons does not prevent their death. **(A)** Immunostaining of c-Fos (magenta), p53 (green), and ChAT+ (blue) of L1 motor neurons (MNs) from P4 control wild-type (WT), *SMN*Δ*7* mutant (SMA) and control (WT *c-fos^Δ^ *^MN^**) or *SMN*Δ*7* mutant (*c-fos^Δ^ *^MN^**) with MN-specific c-Fos knockout, respectively. Scale bar = 50 μm. **(B)** Immunostaining of ChAT + (green) L1 MNs of P10 mice from the same group as in panel **(A)**. Scale bar = 50 μm. Quantification of c-Fos **(C)**, p53 **(D)** positive L1 MNs in% and L1 MN numbers **(E)** from the same group as in panel **(A)**. **(F)** Quantification of L1 MN number from P10 mice from the same group as in panel **(A)** plus AAV9-Cre (induce postnatal c-Fos deletion in MNs) or AAV9-GFP (control virus) injected *SMN*Δ*7* mice containing homozygous floxed c-Fos genes (SMA *c-fos*^fl/fl^**). *n* = 3–5 per genotype. Statistics: Kruskal–Wallis test for panels **(C,D)**, one-way ANOVA with Tukey’s correction for panels **(E,F)**. Each data point (n) represents one animal. Asterisks on top of bars without horizontal line indicate the significance compared to another group. **p < 0.01; ***p < 0.001.

So far, we established that p53-dependent c-Fos expression marks vulnerable motor neurons in the severe *SMN*Δ*7* SMA mouse model. Next, we sought out to determine whether c-Fos also causes motor neuron death. To do so, we generated a *SMN*Δ*7* mouse line with an embryonic motor neuron-specific *c-fos* knock-out using the *ChAT-Cre*; *c-fos* loxP system (SMA *c-fos^Δ^ *^MN^**; WT *c-fos^Δ^ *^MN^**) (Fleischmann et al., 2003; Rossi et al., 2011). SMA *c-fos^Δ^ *^MN^** and WT *c-fos^Δ^ *^MN^** lack a complete expression of c-Fos in motor neurons compared to littermates without expressing ChAT-Cre (SMA *c-fos*^fl/fl^** = SMA; WT *c-fos*^fl/fl^** = WT) (Figures 3A,C), while other spinal neurons remain c-Fos positive (Supplementary Figure 3A), confirming a motor neuron-specific c-Fos deletion. In agreement with our previous findings of c-Fos being downstream of p53 (Figure 2), p53 expression was not altered in motor neurons after c-Fos deletion (Figure 3D). Complete motor neuron-specific c-Fos deletion neither delayed nor prevented motor neuron death in P4 or P10 SMA *c-fos^Δ^ *^MN^** mice and also did not alter motor neuron numbers in WT *c-fos^Δ^ *^MN^** littermates (Figures 3B,E,F). To exclude the possibility that another p53 effector executes motor neuron death as a homeostatic response after embryonic c-Fos deletion, we postnatally knocked down c-Fos in SMA *c-fos*^fl/fl^** by ICV injection of a virus expressing Cre recombinase (AAV9-Cre) or GFP (AAV9-GFP) as control, respectively. Although we successfully depleted c-Fos in almost all motor neurons (Supplementary Figure 3), their death was not prevented in SMA *c-fos*^fl/fl^** + Cre (Figure 3F). Taken together, these results demonstrate that c-Fos does not mediate motor neuron death in SMA mice.

### Motor neuron-specific c-Fos deletion does not affect sensory-motor circuit in wild-type and spinal muscular atrophy mice

**FIGURE 4:**
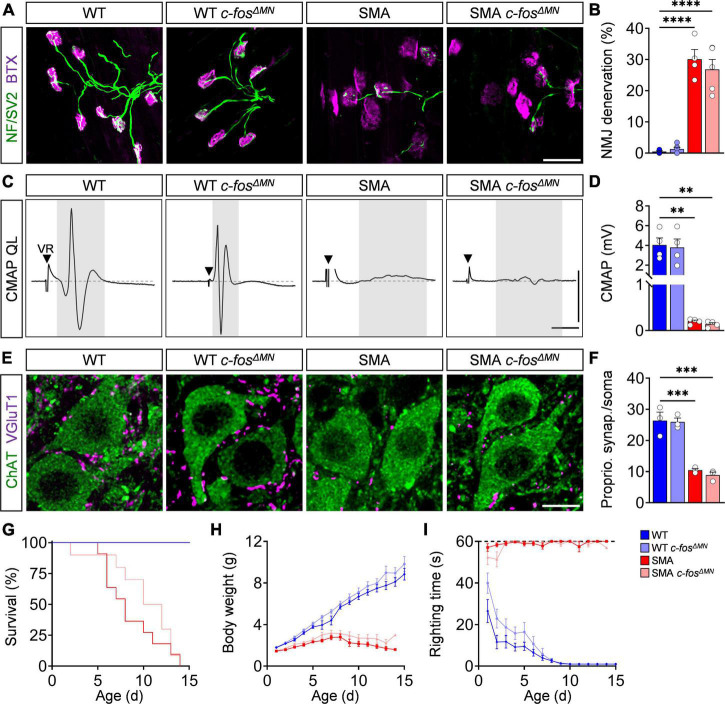
c-Fos deletion in motor neurons has no impact on spinal muscular atrophy (SMA) spinal motor circuits and phenotype. **(A)** Neuromuscular junction (NMJ) staining with bungarotoxin (BTX, magenta), SV2 and neurofilament-M (both green) in the quadratus lumborum (QL) muscles P10 control wild-type (WT), *SMN*Δ*7* mutant (SMA) and control (WT *c-fos^Δ^ *^MN^**) or *SMN*Δ*7* mutant (*c-fos^Δ^ *^MN^**) with MN-specific c-Fos knockout, respectively. Scale bar = 50 μm. **(B)** Percentage of NMJ denervation of the QL muscle from the same groups as in panel **(A)**. *n* = 4–7 per genotype. **(C)** Compound muscle action potential (CMAP) recordings from the QL muscle following L1 ventral root (VR) stimulation from the same groups as in panel **(A)** at P10. Black triangles indicate stimulation artifact and gray box labels CMAP response. Scale bars = 1.5 mV, 3 ms. **(D)** Quantification of CMAP amplitude in mV recorded from the QL muscle in the same groups as in panel **(A)** at P10. *n* = 4 per genotype. **(E)** Immunostaining of VGluT1+ synapses (magenta) and ChAT + L1 motor neurons (green) from the same groups as in panel **(A)**. Scale bar = 10 μm. **(F)** Number of VGluT1 + synapses on L1 motor neuron somata from the same groups as in panel **(A)**. *n* = 3 per genotype. **(G–I)** Survival, body weight and righting time for the same groups as in panel **(A)**. *n* = 9–12 per genotype. Statistics: one-way ANOVA with Tukey’s correction for panels **(B,D,F)**, Mantel–Cox test for survival **(G)**, multiple *t*-test with Holm–Sidak method for body weight **(H)** and righting time **(I)**. Each data point (n) represents one animal. Asterisks on top of bars without horizontal line indicate the significance compared to another group. **p < 0.01; ***p < 0.001, ****p < 0.0001.

Although c-Fos does not play a role in motor neuron degeneration, its expression has been associated with axonal plasticity of the NMJ as functional output of the sensory-motor circuit (Sanyal et al., 2003; Akiyama et al., 2019), which is heavily affected in SMA (Mentis et al., 2011; Imlach et al., 2012; Lotti et al., 2012; Torres-Benito et al., 2012; Fletcher et al., 2017; Buettner et al., 2021; Lopez-Manzaneda et al., 2021). Therefore, we studied the effect of motor neuron-specific c-Fos deletion on NMJ pathology by visualizing the pre- and postsynaptic part with antibodies against neurofilament/SV2 and BTX, respectively. As reported previously (Simon et al., 2017, 2021; Buettner et al., 2021), the axial muscle QL innervated by proximal L1–L3 motor neurons of P10 SMA mice expressing c-Fos (SMA *c-fos*^fl/fl^** = SMA) was strongly denervated compared to control mice (WT *c-fos*^fl/fl^** = WT) (Figures 4A,B). However, deletion of c-Fos did not alter the degree of NMJ denervation in SMA and WT mice (SMA *c-fos^Δ^ *^MN^**; WT *c-fos^Δ^ *^MN^**) (Figures 4A,B). To access NMJ function, we stimulated the ventral root L1 to record the CMAP and conduction velocity (CV) of the QL muscle in an *ex vivo* preparation, which are both heavily reduced in SMA mice (Figures 4C,D; Supplementary Figure 4A; Simon et al., 2017; Kong et al., 2021). c-Fos knockout had no effect on NMJ function and CV in SMA *c-fos^Δ^ *^MN^** and WT *c-fos^Δ^ *^MN^** mice (Figures 4C,D and Supplementary Figure 4A), demonstrating the lack of cell-autonomous c-Fos impact on NMJ innervation and function. Next, we evaluated the number of VGlut1+ proprioceptive synapses onto vulnerable L1 motor neurons. Similar to the results of the NMJ, c-Fos does not impact the number of proprioceptive synapses on motor neuron soma and dendrites of SMA *c-fos^Δ^ *^MN^** and WT *c-fos^Δ^ *^MN^** mice (Figures 4E,F and Supplementary Figure 4B). In agreement with the lack of c-Fos function in sensory-motor circuit, c-Fos absence in motor neurons only insignificantly extended survival of SMA mice, but did not impact body weight nor motor behavior assessed by the righting reflex of SMA *c-fos^Δ^ *^MN^** and WT *c-fos^Δ^ *^MN^** animals compared to c-Fos expressing littermates (Figures 4G–I). In conclusion, c-Fos knockout in motor neurons had no effect on the sensory-motor circuit and motor function of SMA or WT mice.

### c-Fos upregulation marks motor neuron death in an intermediate spinal muscular atrophy mouse model

**FIGURE 5:**
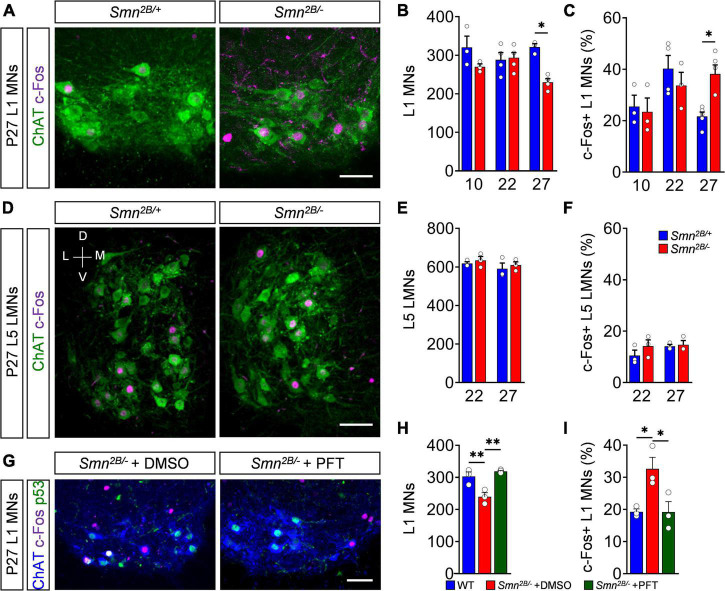
p53-dependent c-Fos upregulation marks motor neuron death in an intermediate spinal muscular atrophy (SMA) model. **(A)** Immunostaining of c-Fos (magenta) and ChAT + (green) L1 motor neurons (MNs) from P27 control (*Smn*^2*B*/+^) and SMA (*Smn^2*B/*–^*) mice. Scale bar = 50 μm. Quantification of L1 **(B)** MN number and **(C)** c-Fos positive MNs in% of P10, P22 and P27 *Smn*^2*B*/+^ and *Smn*^2*B*/+^ mice. *n* = 3–4 per genotype. **(D)** Immunostaining of c-Fos (magenta) and ChAT + (green) L5 lateral motor neurons (LMNs) from P27 *Smn*^2*B*/+^ and SMA *Smn^2*B/*–^* mice. Scale bar = 100 μm. V, ventral; D, dorsal; M, medial; L, lateral. Quantification of L5 LMN **(E)** MN number and **(F)** c-Fos positive MNs in% of P10, P22 and P27 *Smn*^2*B*/+^ and *Smn*^2*B*/–^ mice. *n* = 3 per genotype. **(G)** Immunostaining of c-Fos (magenta), p53 (green) and ChAT + (blue) L1 motor neurons (MNs) from P27 *Smn^2*B/*–^* chronically treated with DMSO or Pifithrin-α (PFT). Scale bar = 100 μm. Quantification of L1 **(H)** MN numbers and **(I)** c-Fos positive MNs in% of P27 *Smn*^2*B*/+^ and *Smn*^2*B*/–^ mice treated with DMSO or PFT. *n* = 3 per genotype. Statistics: two-way ANOVA with Tukey’s correction for panels **(B,C,E,F)**. One-way ANOVA with Tukey’s correction for panels **(H,I)**. Each data point (n) represents one animal. Asterisks on top of bars without horizontal line indicate the significance compared to another group. *p < 0.05; **p < 0.01.

Finally, we inquired whether c-Fos activation in degenerating motor neurons is limited to this severe model or conserved in different forms of SMA. Therefore, we correlated the time course of c-Fos expression with motor neuron death in *Smn^2*B/*–^* mice, which models an intermediate form of SMA with a life span of about 4 weeks (Bowerman et al., 2012). *Smn^2*B/*–^* mutant mice in the *C57BL6* background exhibited a very late, selective death of L1 motor neurons at end-stage (P27), while no loss at P10 and P22 was detected compared to *Smn*^2*B*/+^ control littermates (Figures 5A,B), as previously reported (Buettner et al., 2021). Nuclear c-Fos expression in L1 motor neurons of *Smn^2*B/*–^* mice was similar to control levels at P10 and P22 (Figures 5A,C). Strikingly, c-Fos expression doubles in L1 motor neurons at P27 (Figures 5A,C), while we did not observe any significant change in non-motor neuron cells in the L1 spinal segment (Supplementary Figures 5A,B) or resistant L5 LMNs of end-stage *Smn^2*B/*–^* mice (Figures 5D–F). These observations correlate the nuclear c-Fos upregulation with the death of vulnerable motor neurons in this SMA mouse model. Next, we investigated whether c-Fos upregulation is also p53-dependent in this intermediate SMA model. While c-Fos and p53 are co-expressed in motor neurons (Figure 5G), c-Fos did not colocalize with p53 outside of the motor neuron pool of end-stage *Smn^2*B/*–^* mice (Supplementary Figure 5C). To investigate whether c-Fos upregulation is also p53-dependent, we chronically treated *Smn^2*B/*–^* mice with PFT that rescued L1 motor neurons by inhibition of p53 without altering its expression (Figures 5G,H), as reported previously (Buettner et al., 2021). Importantly, p53 inhibition decreased nuclear c-Fos in motor neurons of *Smn^2*B/*–^* mice to control levels (Figure 5I), demonstrating a p53-dependent c-Fos expression in an intermediate SMA mouse model. Interestingly, the increased proportion of vulnerable SMA L1 motor neurons expressing nuclear c-Fos protein correlates with the timing of their loss in this intermediate model. Taken together, p53-dependent expression of c-Fos marks degenerating motor neurons of different SMA mouse models.

## Discussion

Our study reveals that c-Fos upregulation correlates with the time course and amount of occurring motor neuron death across different severity forms of SMA. The upregulation of c-Fos in SMA is induced by the activation of the p53 pathway. The fact that c-Fos deletion in motor neurons did not improve neuronal death or the phenotype of SMA mice indicates a non-pathogenic role of c-Fos. However, the identification of c-Fos as a marker for neuronal death could be a valuable readout in p53-dependent neurodegeneration for novel therapeutic approaches.

In this study, we identify p53-dependent c-Fos upregulation as a marker for degenerating motor neurons in mouse models of SMA with different severity forms. In our control and SMA mice, baseline c-Fos expression fluctuates during the time course of development. In striking contrast, an additional subset of motor neurons in mutants of both mouse lines express c-Fos, which correlates with the time of onset of motor neuron death in SMA, raising the question for the mechanism of additional c-Fos expression in vulnerable motor neurons. c-Fos binds to members of the Fos or Jun family to form the transcription factor activator protein-1, which acts in a wide range of cellular processes, including cell growth, differentiation, neuronal death, and synaptic function (Smeyne et al., 1993; Hafezi et al., 1997; Elkeles et al., 1999; Ameyar et al., 2003; Sanyal et al., 2003; Xu et al., 2005). While c-Fos has been postulated to participate in molecular mechanisms of long-term memory and normal brain development (Fleischmann et al., 2003; Velazquez et al., 2015), its best known function is a proxy of neuronal activity due to its expression upon depolarization induced by activity-enhancing drugs or proprioceptive activation onto motor neurons (Hunt et al., 1987; Dragunow and Faull, 1989; Simon et al., 2021). Although SMA motor neurons have been reported to be hyperexcitable (Mentis et al., 2011; Simon et al., 2016; Fletcher et al., 2017), it is unlikely that increased neuronal activity is responsible for the changes of c-Fos expression in healthy motor neurons or degenerating SMA motor neurons during development. On the one hand, hyperexcitability of SMA motor neurons does not compensate for the vast reduction of excitation caused by selective loss and dysfunction of excitatory synapses from proprioceptive neurons and other interneurons, while inhibition is unaffected (Mentis et al., 2011; Imlach et al., 2012; Simon et al., 2016; Fletcher et al., 2017). On the other hand, the resting potential of healthy motor neurons remains consistent during postnatal development and is unaltered in motor neurons of SMA mouse models (Mentis et al., 2011; Quinlan et al., 2019; Smith and Brownstone, 2020). This indicates that c-Fos upregulation is independent of neuronal activity, which is in agreement with our findings in this study that c-Fos upregulation in motor neurons is driven by p53 activation in SMA mice. c-Fos protein has been identified during transcriptional profiling of vulnerable motor neurons that degenerate through p53 pathway activation in SMA mice (Murray et al., 2015; Simon et al., 2017; Buettner et al., 2021). It has been shown that motor neuron death in SMA mice requires converging mechanisms of upregulation and amino-terminal phosphorylation of p53 (Simon et al., 2017, 2019; Van Alstyne et al., 2018; Buettner et al., 2021; Carlini et al., 2022). SMN-dependent missplicing of Mdm2 and Mdm4, two main negative p53 regulators, induces p53 upregulation while aberrant splicing of Stasimon phosphorylates p53 through the p38 mitogen activated protein kinase (MAPK) (Van Alstyne et al., 2018; Simon et al., 2019). Intriguingly, p53 expression occurs in both, resistant and vulnerable motor neurons prior to c-Fos upregulation in both SMA mouse models. In contrast, the c-Fos upregulation timely correlates with the phosphorylated form of p53 exclusively in degenerating motor neurons (Simon et al., 2017; Buettner et al., 2021). This and the fact that pharmacological inhibition of p53 transcriptional activity prevents motor neuron death and reduces c-Fos to control levels, without altering p53 levels itself suggests that p53 upregulation alone is insufficient and requires further modifications (e.g., phosphorylation) to induce SMA specific c-Fos expression that serves as a marker for motor neuron death across mouse models.

Previous studies show that c-Fos and other members of the Fos family can induce neuronal death in a p53-dependent manner (Preston et al., 1996; Hafezi et al., 1997; Chen et al., 2015), modulate NMJ function in *Drosophila* (Sanyal et al., 2003) and induce motor axon branching in human ALS motor neurons (Akiyama et al., 2019). To decipher the function of c-Fos in SMA motor neuron pathology with the hallmarks of cellular death and NMJ denervation, we deleted c-Fos specifically in motor neurons. However, we demonstrated in this study that motor-neuron specific c-Fos deletion has no effect on the overall phenotype, motor neuron numbers, NMJ innervation or function of wild-type and SMA mice. In agreement, pharmacological increase of c-Fos does not worsen motor neuron death in SMA mice and accompanies neuroprotection in ALS (Baczyk et al., 2020; Simon et al., 2021). This is in line with c-Fos upregulation consistently marking excitotoxicity-mediated neuronal death in the brain and motor neuron death after sciatic nerve lesion (Smeyne et al., 1993; Xu et al., 2005), but c-Fos deletion does not prevent these degenerative processes (Roffler-Tarlov et al., 1996). Since c-Fos seems to have no pathogenic role in SMA motor neuron death, the executing p53 downstream target has yet to be identified. A recent study of ALS human motor neurons reported that p53 activates a downstream apoptotic program, including Puma, which drives neurodegeneration (Maor-Nof et al., 2021). However, we did not find any evidence for elevated levels of apoptotic DNA fragmentation or cleaved forms of caspases in motor neurons of SMA mice (Simon et al., 2017). In agreement, many classical p53 effectors linked to apoptosis, including *puma*, *bax, dr5, noxa*, and *pidd*, were not upregulated (Simon et al., 2017), suggesting that p53 activation may trigger a caspase-independent form of cell death in SMA motor neurons. In the same study, several p53 effectors have been found upregulated (Simon et al., 2017). The targets *plk2*, *mmp2*, *aldh4a1* and *crip2*, *cdkn1a* (p21) are associated with suppression of apoptosis, tumor growth, and oxidative stress response, but not with cell death (Seoane et al., 2002; Miyake et al., 2015; Han et al., 2018). Another group of targets consisting of *gtse1* (G two S phase expressed protein 1), *ccng1* (cyclin G1), and *sesn1* (sestrin-1) are better known for their functions in cell cycle arrest, while *fas*, *perp* and *c-fos* have been associated with neuronal death (Attardi et al., 2000; Ihrie et al., 2003; Hartland et al., 2009; Sareen et al., 2012; Murray et al., 2015; Simon et al., 2017). Our FISH analysis deciphers an exclusive time correlation of *c-fos* mRNA upregulation with motor neuron death, while *perp* and *fas* increase throughout the disease progression even when no further degeneration occurs. However, it cannot be ruled out that Perp or Fas contribute to p53-dependent motor neuron death. A recent study discovered a contribution of B-Raf signaling to motor neuron degeneration in SMA (Hensel et al., 2021). B-Raf signaling pathway directly interacts with p53 and regulates p38 MAPK activity (Xing et al., 2000; Falcicchio et al., 2020) which is required to mediate p53-dependent motor neuron death in SMA mice (Simon et al., 2017, 2019), prompting speculation that members of B-Raf signaling pathway could act as p53 executors. Overall, our findings rule out c-Fos as a candidate executioner of p53-mediated death of motor neurons and other aspects of SMA pathology. Nevertheless, they point to c-Fos as a p53-dependent marker of vulnerable SMA motor neurons and a potential diagnostic readout in SMA and possibly other p53-associated neurodegenerative diseases.

## Data availability statement

The original contributions presented in this study are included in the article/Supplementary material, further inquiries can be directed to the corresponding author.

## Ethics statement

The animal study was reviewed and approved by the regional directorate (Landesdirektion) of Leipzig.

## Author contributions

CMS designed and supervised the study. JMB, LS, FG, and BB-R performed the experiments and analyzed the data. SH provided valuable input to the interpretation of the results. CMS wrote the manuscript with input from all authors. All authors have read and approved the final manuscript.

## References

[B1] Akiyama T. Suzuki N. Ishikawa M. Fujimori K. Sone T. Kawada J. (2019). Aberrant axon branching via Fos-B dysregulation in FUS-ALS motor neurons. *EBioMedicine* 45 362–378. 10.1016/j.ebiom.2019.06.013 31262712 PMC6642224

[B2] Ameyar M. Wisniewska M. Weitzman J. B. (2003). A role for AP-1 in apoptosis: The case for and against. *Biochimie* 85 747–752. 10.1016/j.biochi.2003.09.006 14585541

[B3] Attardi L. D. Reczek E. E. Cosmas C. Demicco E. G. McCurrach M. E. Lowe S. W. (2000). PERP, an apoptosis-associated target of p53, is a novel member of the PMP-22/gas3 family. *Genes Dev.* 14 704–718. 10733530 PMC316461

[B4] Baczyk M. Alami N. O. Delestree N. Martinot C. Tang L. Commisso B. (2020). Synaptic restoration by cAMP/PKA drives activity-dependent neuroprotection to motoneurons in ALS. *J. Exp. Med.* 217:e20191734. 10.1084/jem.20191734 32484501 PMC7398175

[B5] Bano D. Zanetti F. Mende Y. Nicotera P. (2011). Neurodegenerative processes in Huntington’s disease. *Cell Death Dis.* 2:e228. 10.1038/cddis.2011.112 PMC3223696 22071633

[B6] Blanco-Redondo B. Nuwal N. Kneitz S. Nuwal T. Halder P. Liu Y. (2019). Implications of the Sap47 null mutation for synapsin phosphorylation, longevity, climbing proficiency and behavioural plasticity in adult *Drosophila*. *J. Exp. Biol.* 222:jeb203505. 10.1242/jeb.203505 31488622

[B7] Bowerman M. Murray L. M. Beauvais A. Pinheiro B. Kothary R. (2012). A critical smn threshold in mice dictates onset of an intermediate spinal muscular atrophy phenotype associated with a distinct neuromuscular junction pathology. *Neuromuscul. Disord.* 22 263–276. 10.1016/j.nmd.2011.09.007 22071333

[B8] Buettner J. M. Kirmann T. Mentis G. Z. Hallermann S. Simon C. M. (2022). Laser microscopy acquisition and analysis of premotor synapses in the murine spinal cord. *STAR Protoc.* 3:101236. 10.1016/j.xpro.2022.101236 35300003 PMC8920933

[B9] Buettner J. M. Sime Longang J. K. Gerstner F. Apel K. S. Blanco-Redondo B. Sowoidnich L. (2021). Central synaptopathy is the most conserved feature of motor circuit pathology across spinal muscular atrophy mouse models. *iScience* 24:103376. 10.1016/j.isci.2021.103376 34825141 PMC8605199

[B10] Burghes A. H. Beattie C. E. (2009). Spinal muscular atrophy: Why do low levels of survival motor neuron protein make motor neurons sick? *Nat. Rev. Neurosci.* 10 597–609. 10.1038/nrn2670 19584893 PMC2853768

[B11] Carlini M. J. Triplett M. K. Pellizzoni L. (2022). Neuromuscular denervation and deafferentation but not motor neuron death are disease features in the Smn2B/- mouse model of SMA. *PLoS One* 17:e0267990. 10.1371/journal.pone.0267990 35913953 PMC9342749

[B12] Chang J. R. Ghafouri M. Mukerjee R. Bagashev A. Chabrashvili T. Sawaya B. E. (2012). Role of p53 in neurodegenerative diseases. *Neurodegener. Dis.* 9 68–80. 22042001 10.1159/000329999 PMC3304514

[B13] Chen X. Shen J. Wang Y. Chen X. Yu S. Shi H. (2015). Up-regulation of c-Fos associated with neuronal apoptosis following intracerebral hemorrhage. *Cell. Mol. Neurobiol.* 35 363–376. 10.1007/s10571-014-0132-z 25354492 PMC11486182

[B14] Dai C. Gu W. (2010). p53 post-translational modification: Deregulated in tumorigenesis. *Trends Mol. Med.* 16 528–536. 20932800 10.1016/j.molmed.2010.09.002 PMC2978905

[B15] Donev R. Kolev M. Millet B. Thome J. (2009). Neuronal death in Alzheimer’s disease and therapeutic opportunities. *J. Cell. Mol. Med.* 13 4329–4348. 19725918 10.1111/j.1582-4934.2009.00889.x PMC4515050

[B16] Dragunow M. Faull R. (1989). The use of c-fos as a metabolic marker in neuronal pathway tracing. *J. Neurosci. Methods* 29 261–265. 10.1016/0165-0270(89)90150-7 2507830

[B17] Elkeles A. Juven-Gershon T. Israeli D. Wilder S. Zalcenstein A. Oren M. (1999). The c-fos proto-oncogene is a target for transactivation by the p53 tumor suppressor. *Mol. Cell. Biol.* 19 2594–2600. 10.1128/MCB.19.4.2594 10082525 PMC84052

[B18] Falcicchio M. Ward J. A. Macip S. Doveston R. G. (2020). Regulation of p53 by the 14-3-3 protein interaction network: New opportunities for drug discovery in cancer. *Cell Death Discov.* 6:126. 10.1038/s41420-020-00362-3 33298896 PMC7669891

[B19] Feldkotter M. Schwarzer V. Wirth R. Wienker T. F. Wirth B. (2002). Quantitative analyses of SMN1 and SMN2 based on real-time lightCycler PCR: Fast and highly reliable carrier testing and prediction of severity of spinal muscular atrophy. *Am. J. Hum. Genet.* 70 358–368. 10.1086/338627 11791208 PMC419987

[B20] Fleischmann A. Hvalby O. Jensen V. Strekalova T. Zacher C. Layer L. E. (2003). Impaired long-term memory and NR2A-type NMDA receptor-dependent synaptic plasticity in mice lacking c-Fos in the CNS. *J. Neurosci.* 23 9116–9122. 10.1523/JNEUROSCI.23-27-09116.2003 14534245 PMC6740829

[B21] Fletcher E. V. Simon C. M. Pagiazitis J. G. Chalif J. I. Vukojicic A. Drobac E. (2017). Reduced sensory synaptic excitation impairs motor neuron function via Kv2.1 in spinal muscular atrophy. *Nat. Neurosci.* 20 905–916. 10.1038/nn.4561 28504671 PMC5487291

[B22] Hafezi F. Steinbach J. P. Marti A. Munz K. Wang Z. Q. Wagner E. F. (1997). The absence of c-fos prevents light-induced apoptotic cell death of photoreceptors in retinal degeneration in vivo. *Nat. Med.* 3 346–349. 10.1038/nm0397-346 9055866

[B23] Han T. Lin J. Wang Y. Fan Q. Sun H. Tao Y. (2018). Forkhead box D1 promotes proliferation and suppresses apoptosis via regulating polo-like kinase 2 in colorectal cancer. *Biomed. Pharmacother.* 103 1369–1375. 29864920 10.1016/j.biopha.2018.04.190

[B24] Hartland S. N. Murphy F. Aucott R. L. Abergel A. Zhou X. Waung J. (2009). Active matrix metalloproteinase-2 promotes apoptosis of hepatic stellate cells via the cleavage of cellular N-cadherin. *Liver Int.* 29 966–978. 10.1111/j.1478-3231.2009.02070.x 19580633

[B25] Hensel N. Cieri F. Santonicola P. Tapken I. Schuning T. Taiana M. (2021). Impairment of the neurotrophic signaling hub B-Raf contributes to motoneuron degeneration in spinal muscular atrophy. *Proc. Natl. Acad. Sci. U.S.A.* 118:e2007785118. 10.1073/pnas.2007785118 33931501 PMC8106332

[B26] Hunt S. P. Pini A. Evan G. (1987). Induction of c-fos-like protein in spinal cord neurons following sensory stimulation. *Nature* 328 632–634. 10.1038/328632a0 3112583

[B27] Ihrie R. A. Reczek E. Horner J. S. Khachatrian L. Sage J. Jacks T. (2003). Perp is a mediator of p53-dependent apoptosis in diverse cell types. *Curr. Biol.* 13 1985–1990. 14614825 10.1016/j.cub.2003.10.055

[B28] Imlach W. L. Beck E. S. Choi B. J. Lotti F. Pellizzoni L. McCabe B. D. (2012). SMN is required for sensory-motor circuit function in *Drosophila*. *Cell* 151 427–439. 10.1016/j.cell.2012.09.011 23063130 PMC3475188

[B29] Kong L. Valdivia D. O. Simon C. M. Hassinan C. W. Delestree N. Ramos D. M. (2021). Impaired prenatal motor axon development necessitates early therapeutic intervention in severe SMA. *Sci. Transl. Med.* 13:eabb6871. 10.1126/scitranslmed.abb6871 33504650 PMC8208236

[B30] Le T. T. Pham L. T. Butchbach M. E. Zhang H. L. Monani U. R. Coovert D. D. (2005). SMNDelta7, the major product of the centromeric survival motor neuron (SMN2) gene, extends survival in mice with spinal muscular atrophy and associates with full-length SMN. *Hum. Mol. Genet.* 14 845–857. 15703193 10.1093/hmg/ddi078

[B31] Lopez-Manzaneda M. Franco-Espin J. Tejero R. Cano R. Tabares L. (2021). Calcium is reduced in presynaptic mitochondria of motor nerve terminals during neurotransmission in SMA mice. *Hum. Mol. Genet.* 30 629–643. 10.1093/hmg/ddab065 33693569 PMC8127408

[B32] Lotti F. Imlach W. L. Saieva L. Beck E. S. Hao le T. Li D. K. (2012). An SMN-dependent U12 splicing event essential for motor circuit function. *Cell* 151 440–454. 10.1016/j.cell.2012.09.012 23063131 PMC3474596

[B33] Maor-Nof M. Shipony Z. Lopez-Gonzalez R. Nakayama L. Zhang Y. J. Couthouis J. (2021). p53 is a central regulator driving neurodegeneration caused by C9orf72 poly(PR). *Cell* 184 689–708.e20. 10.1016/j.cell.2020.12.025 33482083 PMC7886018

[B34] Martinez T. L. Kong L. Wang X. Osborne M. A. Crowder M. E. Van Meerbeke J. P. (2012). Survival motor neuron protein in motor neurons determines synaptic integrity in spinal muscular atrophy. *J. Neurosci.* 32 8703–8715. 22723710 10.1523/JNEUROSCI.0204-12.2012 PMC3462658

[B35] Mentis G. Z. Blivis D. Liu W. Drobac E. Crowder M. E. Kong L. (2011). Early functional impairment of sensory-motor connectivity in a mouse model of spinal muscular atrophy. *Neuron* 69 453–467. 10.1016/j.neuron.2010.12.032 21315257 PMC3044334

[B36] Michel P. P. Hirsch E. C. Hunot S. (2016). Understanding dopaminergic cell death pathways in Parkinson disease. *Neuron* 90 675–691. 27196972 10.1016/j.neuron.2016.03.038

[B37] Miyake M. Goodison S. Lawton A. Gomes-Giacoia E. Rosser C. J. (2015). Angiogenin promotes tumoral growth and angiogenesis by regulating matrix metallopeptidase-2 expression via the ERK1/2 pathway. *Oncogene* 34 890–901. 10.1038/onc.2014.2 24561529 PMC4317372

[B38] Murray L. M. Beauvais A. Gibeault S. Courtney N. L. Kothary R. (2015). Transcriptional profiling of differentially vulnerable motor neurons at pre-symptomatic stage in the Smn (2b/-) mouse model of spinal muscular atrophy. *Acta Neuropathol. Commun.* 3:55. 10.1186/s40478-015-0231-1 26374403 PMC4570693

[B39] Nichterwitz S. Nijssen J. Storvall H. Schweingruber C. Comley L. H. Allodi I. (2020). LCM-seq reveals unique transcriptional adaptation mechanisms of resistant neurons and identifies protective pathways in spinal muscular atrophy. *Genome Res.* 30 1083–1096. 10.1101/gr.265017.120 32820007 PMC7462070

[B40] Preston G. A. Lyon T. T. Yin Y. Lang J. E. Solomon G. Annab L. (1996). Induction of apoptosis by c-Fos protein. *Mol. Cell. Biol.* 16 211–218. 8524298 10.1128/mcb.16.1.211 PMC230994

[B41] Qi X. Davis B. Chiang Y. H. Filichia E. Barnett A. Greig N. H. (2016). Dopaminergic neuron-specific deletion of p53 gene is neuroprotective in an experimental Parkinson’s disease model. *J. Neurochem.* 138 746–757. 10.1111/jnc.13706 27317935 PMC5155515

[B42] Quinlan K. A. Reedich E. J. Arnold W. D. Puritz A. C. Cavarsan C. F. Heckman C. J. (2019). Hyperexcitability precedes motoneuron loss in the Smn(2B/-) mouse model of spinal muscular atrophy. *J. Neurophysiol.* 122 1297–1311. 10.1152/jn.00652.2018 31365319 PMC6843095

[B43] Roffler-Tarlov S. Brown J. J. Tarlov E. Stolarov J. Chapman D. L. Alexiou M. (1996). Programmed cell death in the absence of c-Fos and c-Jun. *Development* 122 1–9. 8565820 10.1242/dev.122.1.1

[B44] Rossi J. Balthasar N. Olson D. Scott M. Berglund E. Lee C. E. (2011). Melanocortin-4 receptors expressed by cholinergic neurons regulate energy balance and glucose homeostasis. *Cell Metab.* 13 195–204. 10.1016/j.cmet.2011.01.010 21284986 PMC3033043

[B45] Sanyal S. Narayanan R. Consoulas C. Ramaswami M. (2003). Evidence for cell autonomous AP1 function in regulation of *Drosophila* motor-neuron plasticity. *BMC Neurosci.* 4:20. 10.1186/1471-2202-4-20 12969508 PMC201019

[B46] Sareen D. Ebert A. D. Heins B. M. McGivern J. V. Ornelas L. Svendsen C. N. (2012). Inhibition of apoptosis blocks human motor neuron cell death in a stem cell model of spinal muscular atrophy. *PLoS One* 7:e39113. 10.1371/journal.pone.0039113 22723941 PMC3378532

[B47] Seoane J. Le H. V. Massague J. (2002). Myc suppression of the p21(Cip1) Cdk inhibitor influences the outcome of the p53 response to DNA damage. *Nature* 419 729–734. 10.1038/nature01119 12384701

[B48] Simon C. M. Blanco-Redondo B. Buettner J. M. Pagiazitis J. G. Fletcher E. V. Sime Longang J. K. (2021). Chronic pharmacological increase of neuronal activity improves sensory-motor dysfunction in spinal muscular atrophy mice. *J. Neurosci.* 41 376–389. 10.1523/JNEUROSCI.2142-20.2020 33219005 PMC7810663

[B49] Simon C. M. Dai Y. Van Alstyne M. Koutsioumpa C. Pagiazitis J. G. Chalif J. I. (2017). Converging mechanisms of p53 activation drive motor neuron degeneration in spinal muscular atrophy. *Cell Rep.* 21 3767–3780. 10.1016/j.celrep.2017.12.003 29281826 PMC5747328

[B50] Simon C. M. Jablonka S. Ruiz R. Tabares L. Sendtner M. (2010). Ciliary neurotrophic factor-induced sprouting preserves motor function in a mouse model of mild spinal muscular atrophy. *Hum. Mol. Genet.* 19 973–986. 10.1093/hmg/ddp562 20022887

[B51] Simon C. M. Janas A. M. Lotti F. Tapia J. C. Pellizzoni L. Mentis G. Z. (2016). A Stem cell model of the motor circuit uncouples motor neuron death from hyperexcitability induced by SMN deficiency. *Cell Rep.* 16 1416–1430. 10.1016/j.celrep.2016.06.087 27452470 PMC4972669

[B52] Simon C. M. Van Alstyne M. Lotti F. Bianchetti E. Tisdale S. Watterson D. M. (2019). Stasimon contributes to the loss of sensory synapses and motor neuron death in a mouse model of spinal muscular atrophy. *Cell Rep.* 29 3885–3901.e5. 10.1016/j.celrep.2019.11.058 31851921 PMC6956708

[B53] Smeyne R. J. Vendrell M. Hayward M. Baker S. J. Miao G. G. Schilling K. (1993). Continuous c-fos expression precedes programmed cell death in vivo. *Nature* 363 166–169. 10.1038/363166a0 8483500

[B54] Smith C. C. Brownstone R. M. (2020). Spinal motoneuron firing properties mature from rostral to caudal during postnatal development of the mouse. *J. Physiol.* 598 5467–5485. 10.1113/JP280274 32851667 PMC8436765

[B55] Tisdale S. Pellizzoni L. (2015). Disease mechanisms and therapeutic approaches in spinal muscular atrophy. *J. Neurosci.* 35 8691–8700. 26063904 10.1523/JNEUROSCI.0417-15.2015 PMC4461682

[B56] Torres-Benito L. Ruiz R. Tabares L. (2012). Synaptic defects in spinal muscular atrophy animal models. *Dev. Neurobiol.* 72 126–133. 21567981 10.1002/dneu.20912

[B57] Van Alstyne M. Simon C. M. Sardi S. P. Shihabuddin L. S. Mentis G. Z. Pellizzoni L. (2018). Dysregulation of Mdm2 and Mdm4 alternative splicing underlies motor neuron death in spinal muscular atrophy. *Genes Dev.* 32 1045–1059. 10.1101/gad.316059.118 30012555 PMC6075148

[B58] Velazquez F. N. Caputto B. L. Boussin F. D. (2015). c-Fos importance for brain development. *Aging (Albany NY)* 7 1028–1029. 26684501 10.18632/aging.100862 PMC4712328

[B59] Vogt M. A. Ehsaei Z. Knuckles P. Higginbottom A. Helmbrecht M. S. Kunath T. (2018). TDP-43 induces p53-mediated cell death of cortical progenitors and immature neurons. *Sci. Rep.* 8:8097. 10.1038/s41598-018-26397-2 29802307 PMC5970242

[B60] Wang D. B. Kinoshita C. Kinoshita Y. Morrison R. S. (2014). p53 and mitochondrial function in neurons. *Biochim. Biophys. Acta* 1842 1186–1197. 24412988 10.1016/j.bbadis.2013.12.015 PMC4074561

[B61] Wirth B. (2021). Spinal muscular atrophy: In the challenge lies a solution. *Trends Neurosci.* 44 306–322. 10.1016/j.tins.2020.11.009 33423791

[B62] Xing H. Zhang S. Weinheimer C. Kovacs A. Muslin A. J. (2000). 14-3-3 proteins block apoptosis and differentially regulate MAPK cascades. *EMBO J.* 19 349–358. 10.1093/emboj/19.3.349 10654934 PMC305572

[B63] Xu W. Chi L. Xu R. Ke Y. Luo C. Cai J. (2005). Increased production of reactive oxygen species contributes to motor neuron death in a compression mouse model of spinal cord injury. *Spinal Cord* 43 204–213. 10.1038/sj.sc.3101674 15520836

[B64] Zhang Y. McLaughlin R. Goodyer C. LeBlanc A. (2002). Selective cytotoxicity of intracellular amyloid beta peptide1-42 through p53 and Bax in cultured primary human neurons. *J. Cell Biol.* 156 519–529. 10.1083/jcb.200110119 11815632 PMC2173346

