# Supplementary material for: p53-dependent c-Fos expression is a marker but not executor for motor neuron death in spinal muscular atrophy mouse models
Source: Front Cell Neurosci. 2022 Nov 7;16:1038276. doi: 10.3389/fncel.2022.1038276 (PMC9676941; doi:10.3389/fncel.2022.1038276)
Supplement: Supplementary file 1 [file Data_Sheet_1.PDF]

Figure S1

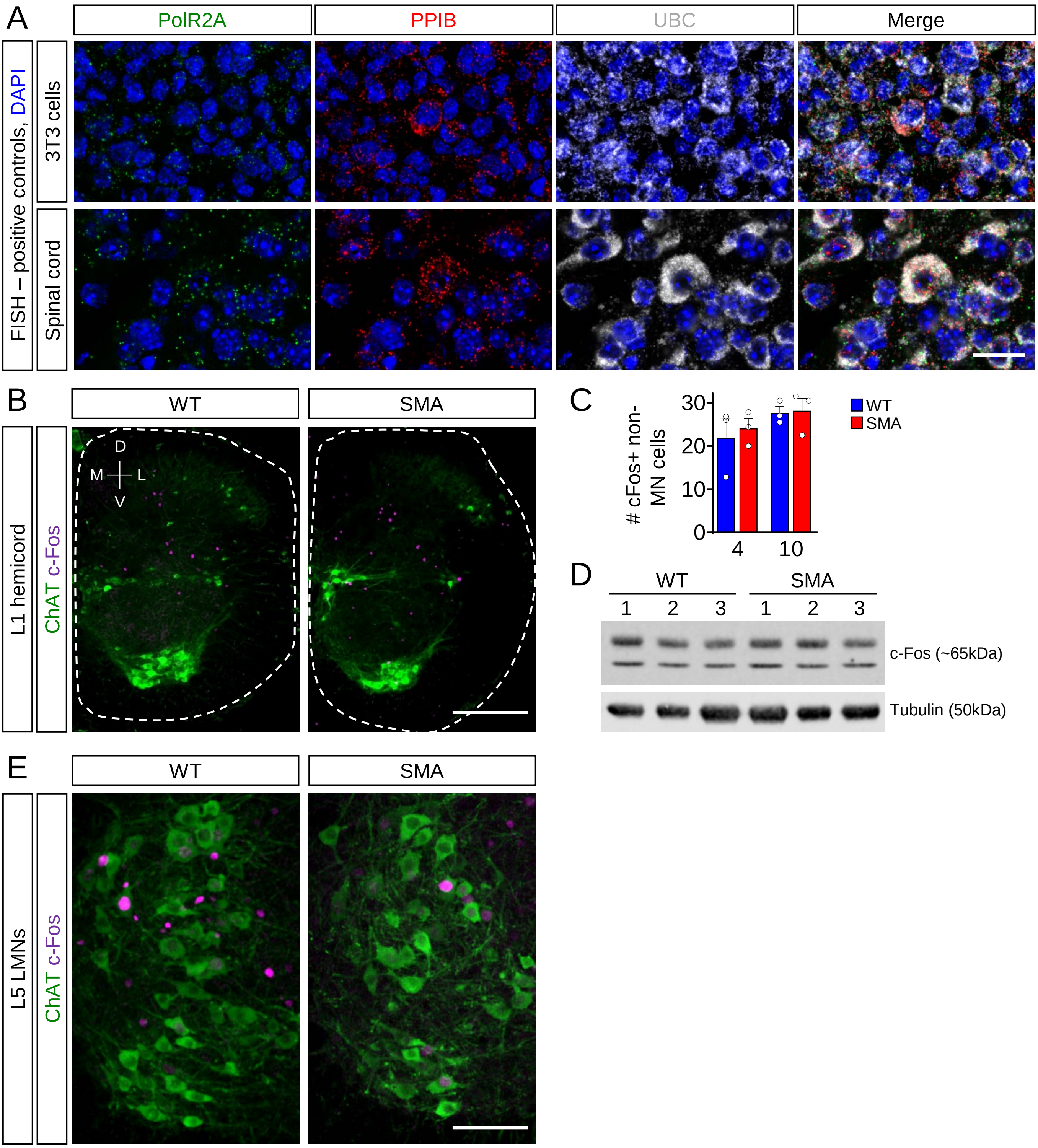

Figure S1. c-Fos protein is only upregulated in degenerating SMA motor neurons.

(A) FISH of three housekeeping genes (PolR2A as low, PPIB as medium and UBC as high mRNA expresser) with DAPI co-staining on 3T3 cells and spinal cord sections as positive controls. Scale bar = 20µm. (B) Immunostaining of c-Fos (magenta) and ChAT (green) of a L1 hemicord from a P10 control (WT) and *SMNΔ7* mutant (SMA) animal. Scale bar = 100µm. V = ventral, D = dorsal, M = medial, L = lateral. (C) Quantification of c-Fos positive non-motor neurons (MNs) of P4 and P10 WT and SMA mice. n = 3 per genotype. (D) Western blot analysis of P4 spinal cord tissue from WT and SMA mice blotted with antibodies against c-Fos and tubulin as a loading control. n = 3 per genotype. (E) Immunostaining of c-Fos (magenta) and ChAT+ (green) L5 lateral motor neurons (LMNs) from P10 WT and SMA mice. Scale bar = 100µm. Statistics: two-way ANOVA with Tukey's correction for C. Each data point (n) represents one animal.

Figure S2

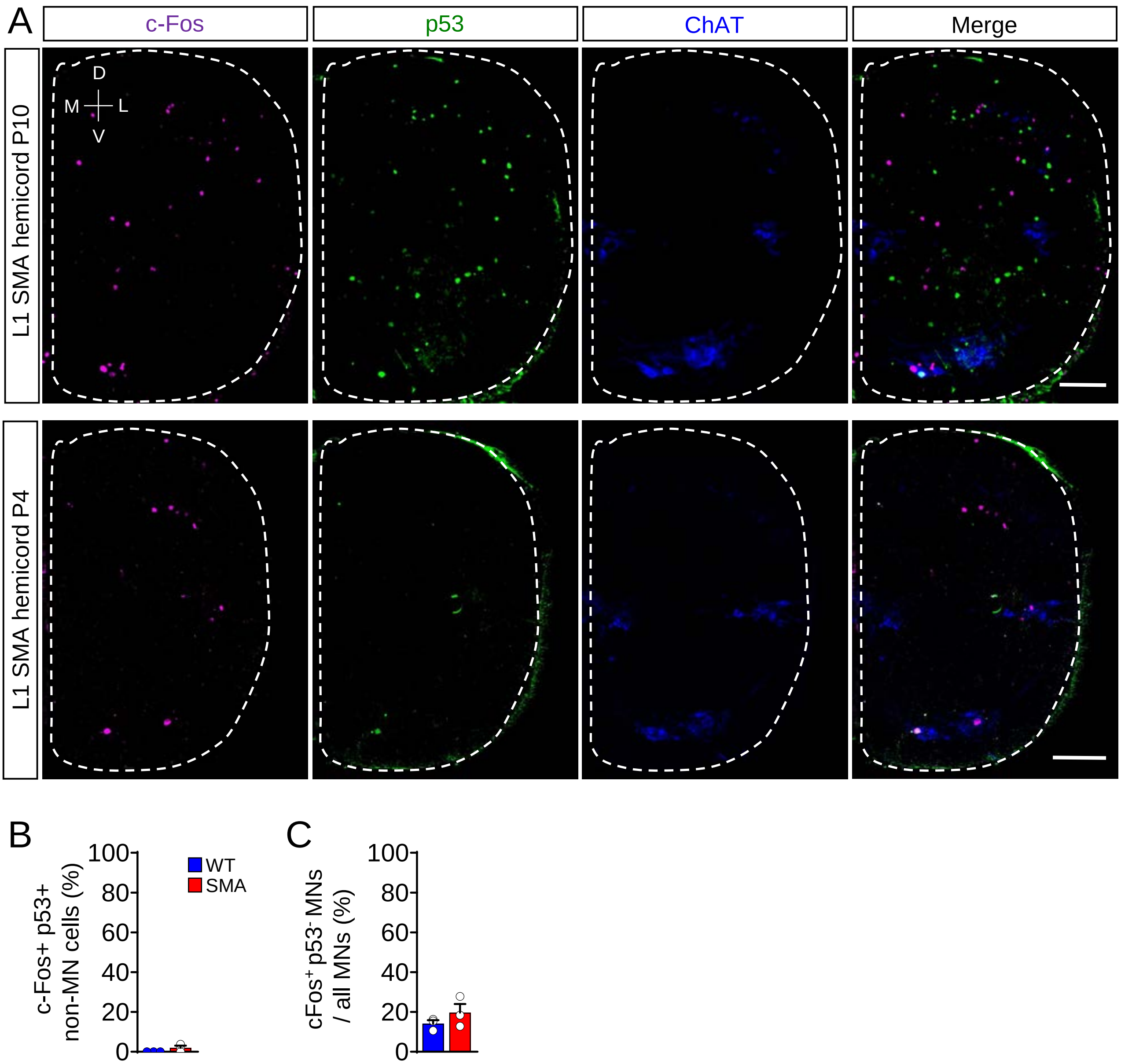

Figure S2. c-Fos and p53 do not colocalize in non-motor neuron cells throughout the spinal cord.

(A) Immunostaining of c-Fos (magenta), p53 (green) and ChAT (blue) on a L1 hemicord from P10 (upper panel) and P4 (lower panel) *SMNΔ7* mutant (SMA) animal. Scale bar = 100µm. V = ventral, D = dorsal, M = medial, L = lateral. (B) Quantification of c-Fos and p53 coexpressing non-motor neurons (MNs) in % throughout P4 WT and SMA spinal cords. n = 3 per genotype. (C) Quantification of c-Fos positive and p53 negative motor neurons compared to all motor neurons of P4 WT and SMA mice in %. n = 3 per genotype. Statistics: Mann-Whitney test for B and two-tailed t-test for C. Each data point (n) represents one animal.

Figure S3

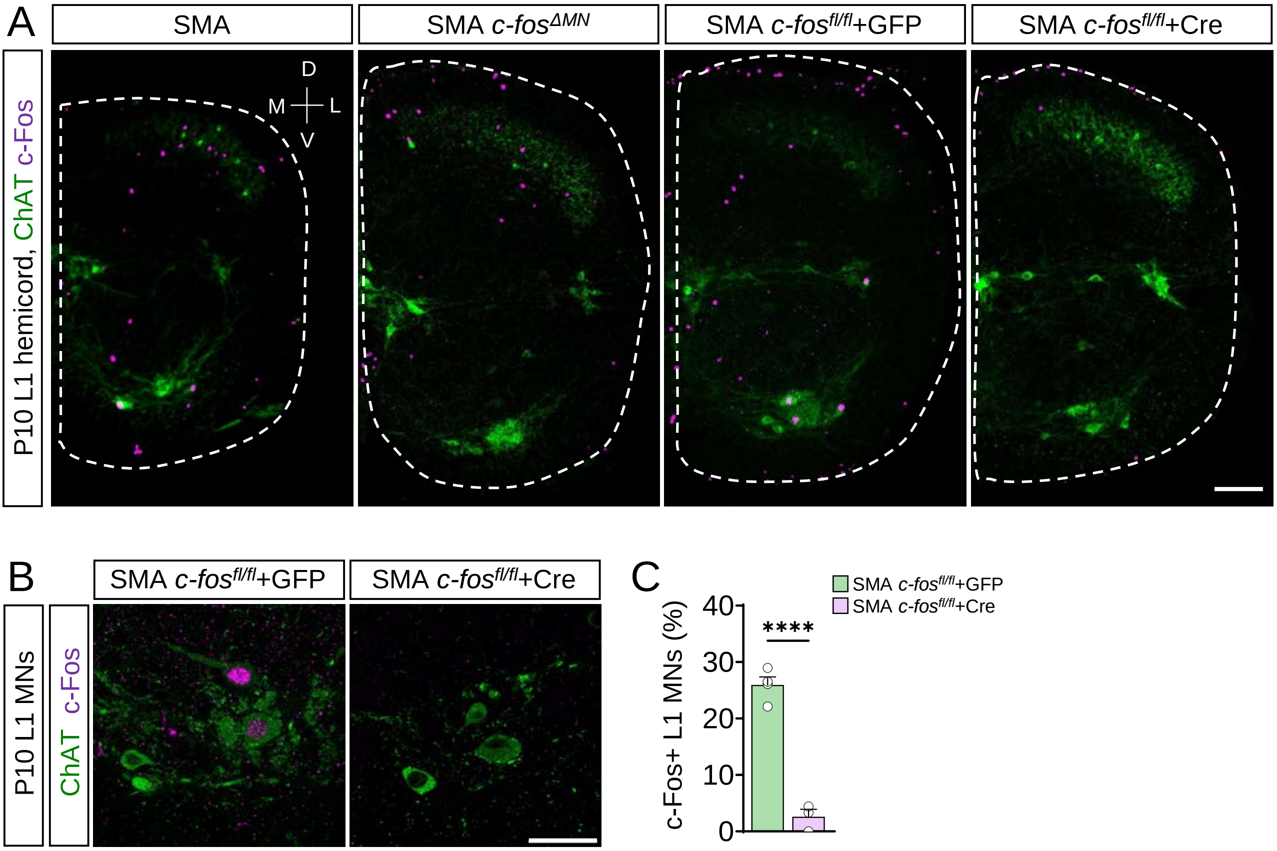

Figure S3. AAV9-Cre efficiently knocksdown c-Fos in motor neurons.

(A) Immunostaining of ChAT and c-Fos of P10 L1 hemicords of *SMNΔ7* mutants (SMA), SMA *c-fos^{ΔMN}* and SMA with homozygous floxed c-Fos genes injected with AAV9-Cre or AAV9-GFP as control. Scale bar = 100µm. V = ventral, D = dorsal, M = medial, L = lateral. (B) Immunostaining of c-Fos (magenta) and ChAT+ (green) L1 motor neurons (MNs) from P10 *SMNΔ7* mutants (SMA) with homozygous floxed c-Fos genes injected with AAV9-Cre or AAV9-GFP as control. Scale bar = 50µm. (C) Quantification of c-Fos positive L1 MNs in % from the same group as in (A). n = 3 per group. Statistics: two-tailed t-test for C. Each data point (n) represents one animal. Asterisks on top of bars without horizontal line indicate the significance compared to another group. ****p<0.0001.

Figure S4

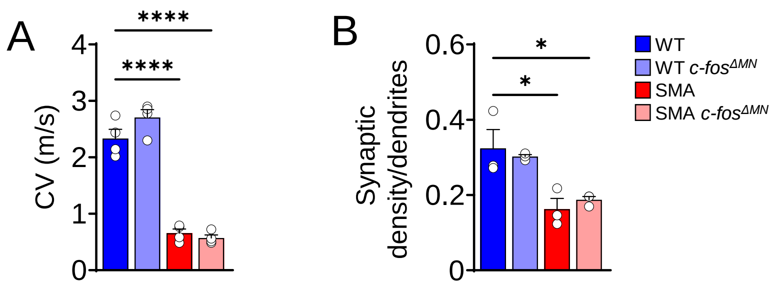

Figure S4. c-Fos knockout does not alter proprioceptive synaptic density and conduction velocity.

(A) Conduction velocity in (m/s) measured between L1 ventral root to quadratus lumborum from P10 control (WT), *SMNΔ7* mutant (SMA) and control (WT *c-fos*$^{\Delta MN}$) or *SMNΔ7* mutant (*c-fos*$^{\Delta MN}$) with MN-specific c-Fos knockout, respectively. n = 4 per genotype. (B) Quantification of VGluT1+ proprioceptive synapses on proximal dendrites (up to 50µm from the motor neuron soma) from the same group as in (A). n = 3-4 per genotype. Statistics: one-way ANOVA with Tukey's correction for A and B. Each data point (n) represents one animal. Asterisks on top of bars without horizontal line indicate the significance compared to another group. *p<0.05; ****p<0.0001.

Figure S5

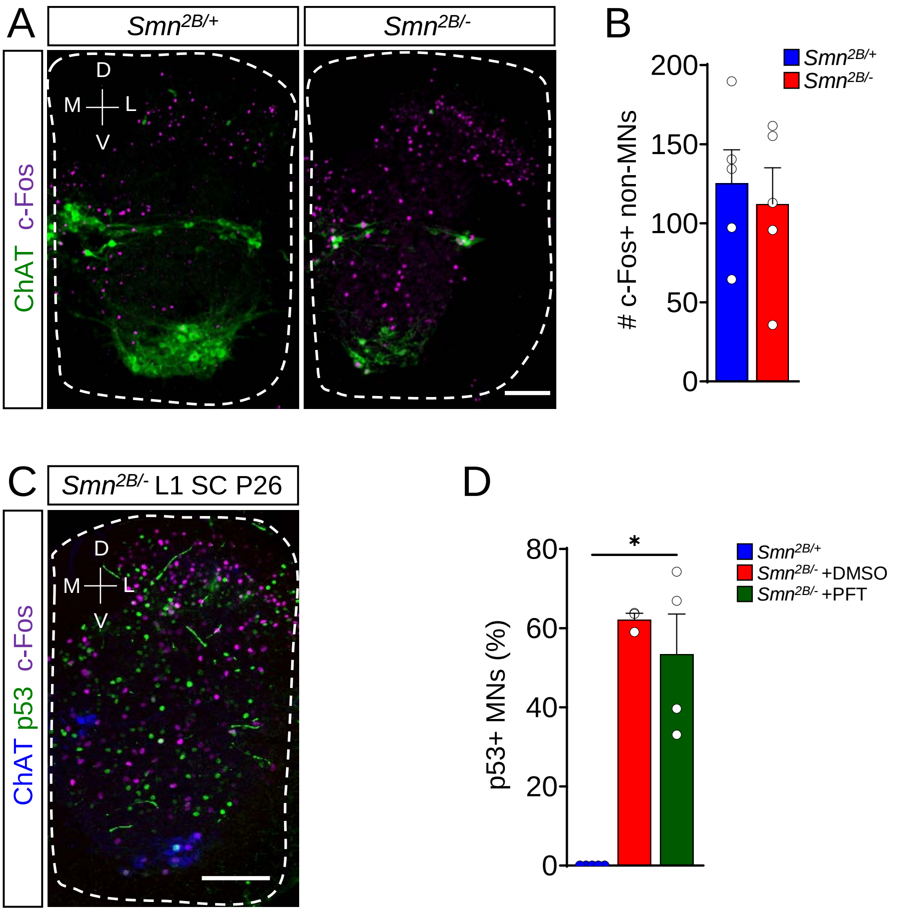

Figure S5. c-Fos is not upregulated in SMA non-motor neuron cells throughout the spinal cord of an intermediate SMA model.

(A) Immunostaining of c-Fos (magenta) and ChAT+ (green) L1 hemicord from P27 control (*Smn$^{2B/+}$*) and SMA (*Smn$^{2B/-}$*) mice. Scale bar = 100µm. V = ventral, D = dorsal, M = medial, L = lateral. (B) Quantification of c-Fos positive non-motor neurons (MNs) of P27 *Smn$^{2B/+}$* and *Smn$^{2B/-}$* mice. n = 5 per genotype. (C) Immunostaining of c-Fos (magenta), p53 (green) and ChAT (blue) on a L1 hemicord from P26 *Smn$^{2B/-}$* mouse. Scale bar = 100µm. (D) Quantification of L1 p53 positive MNs in % of P27 *Smn$^{2B/+}$* and *Smn$^{2B/-}$* mice treated with DMSO or PFT. n = 3-4 per genotype. Statistics: two-tailed t-test for B, Kruskal-Wallis test for D. Each data point (n) represents one animal. Asterisks on top of bars without horizontal line indicate the significance compared to another group. *p<0.05.
